# Study on vertical variation characteristics of soil phosphorus adsorption and desorption in black soil region of Northeast China

**DOI:** 10.1371/journal.pone.0306145

**Published:** 2024-06-24

**Authors:** Wenzhi Zhao, Xu Xie, Tian He, Jintao Zhang, Jiufen Liu

**Affiliations:** 1 Key Laboratory of Coupling Process and Effect of Natural Resources Elements, Beijing, P. R. China; 2 Northeast Geological S&T Innovation Center of China Geological Survey, China Geological Survey, Shenyang, P. R. China; 3 Center for Harbin Natural Resources Comprehensive Survey, China Geological Survey, Harbin, P. R. China; Tennessee State University, UNITED STATES

## Abstract

The adsorption and desorption of phosphorus (P) in soil constitute a crucial internal cycle that is closely associated with soil fertility, exerting direct influence on the quantity, form, and availability of P within the soil. The vertical spatial variation characteristics of soil adsorption and desorption were investigated for the 0–100 cm soil layer in the northeast black soil region in this study. The maximum adsorption capacity (Q_max_) and maximum adsorption buffer capacity (MBC) of black soil in the study area ranged from 313.8 to 411.9 mg kg^-1^ and from 3.1 to 28.8 L kg^-1^, respectively, within the soil layer of 0–100 cm depth, exhibiting an increasing trend with greater soil depth. The degree of P adsorption saturation (DPS) exhibited a contrasting trend with the variations in Q_max_ and MBC, ranging from 3.8% to 21.6%. The maximum desorption capacity (D_max_) and desorption rate (D_r_) of soil P ranged from 112.8 to 215.7 mg kg^-1^ and 32.1% to 52.5%, respectively, while the readily desorbable P (RDP) in soil was within the range of 1.02 to 3.35 mg kg^-1^. Both D_max_, D_r_, and RDP exhibited a decreasing trend with increasing soil depth before showing an upward trend. These research findings not only provide essential background data for the systematic investigation of soil P in the black soil region but also serve as a valuable reference for assessing soil quality in this area.

## Introduction

Phosphorus (P), being one of the indispensable nutrients for plant growth and development, constitutes a crucial component of plant cells [1]. The bioavailability of soil P varies significantly due to its existence in diverse forms [2–4]. The content of bioavailable P that can be directly utilized by plants is relatively limited. A large amount of P is bound in soil minerals, or exists in the form of organoP, which is difficult to be utilized by plants [5–8]. Only soluble H_2_PO_4_^-^ and HPO_4_^2-^ can show biological activity and be absorbed and utilized by plants [9, 10]. The general deficiency of soil available P is an important factor limiting the development of agricultural production in China [11]. The current practice involves the application of P fertilizer to address the issue of insufficient available P in soil, aiming to fulfill plants’ P requirements [12, 13]. However, the utilization efficiency of crops for P fertilizer applied in soil is limited, with a significant portion accumulating as insoluble forms such as calcium phosphate, aluminum phosphate, and iron phosphate. In agricultural soil, insoluble P typically constitutes 60% to 80% of the total P content. The application of P fertilizers can lead to fixation and accumulation in the soil, rendering it unavailable for plant uptake and resulting in significant wastage of P resources [14].

The northeast black soil region serves as a crucial hub for commodity grain production in China [15]. The application of high-concentration phosphate fertilizers, such as superphosphate and ammonium hydrogen phosphate, has been extensively employed in China’s black soil to enhance both crop yield and quality [16]. The average application rate of phosphorus fertilizer in Northeast China is 130.8 kg/hm^2^. The inefficient utilization of P fertilizer results in the accumulation of various forms of P (such as Al-P, Ca-P, and Fe-P) in the soil [17]. Continuous fertilization has the potential to induce alterations in soil properties, encompassing variations in organic matter content, pH levels, and certain biological attributes. These modifications may subsequently impact the soil’s capacity for P absorption [18]. Currently, numerous studies have been conducted to investigate the interactions between different nutrients (such as N, P, and K) and various soil properties across diverse land uses including agriculture, forests, and grasslands [19–23]. Other studies primarily focused on the effects of fertilization [24–27], straw incorporation into the field [28, 29], freeze-thaw cycles [30], autumn burning [31, 32], climate conditions [33, 34] on soil phosphorus content and forms, as well as plant responses to phosphorus restriction [35]. However, little attention has been paid to the adsorption and desorption characteristics of phosphorus in soil [36–41].

The adsorption and desorption of P in soil constitute the pivotal processes governing its behavior, thereby exerting direct influence on the quantity, chemical form, and bioavailability of P within the soil matrix [42]. The adsorption of P compounds by soil represents a primary mechanism through which P is immobilized within the soil matrix. The adsorption process can impose limitations on the utilization of P by plants, whereas desorption can result in the loss of P from the soil. P adsorption encompasses a series of processes (e.g., involving clay minerals and Fe/Al oxides), yet only a fraction of the adsorbed P is accessible to plants [43]. The desorption of soil P is a crucial mechanism for the transfer of P from the solid phase to the liquid phase, contributing significantly to its release in soil.

Currently, research on phosphorus adsorption-desorption in black soil of northeast China primarily focuses on the impacts of various fertilization methods, different small molecular organic acids, and humic acids on surface soil phosphorus adsorption-desorption. However, there is a scarcity of studies investigating soil phosphorus adsorption-desorption at different depths [44–47]. Therefore, this study focuses on investigating the adsorption-desorption characteristics of P in the 0–100 cm soil profile within the black soil region of northeast China. The aim is to provide background data for understanding the distribution of soil P in this region and to establish a theoretical basis for scientifically evaluating the evolution law of black soil quality.

## Materials and methods

### Study area and soil sample collection

The study was conducted at Jinhe Farm, located in Heihe City, Heilongjiang Province, China (latitude 50°15’47" N, longitude 127°26’56" E). The predominant soil type in this region is characterized by dark brown color, classifying it as a variant of black soil. Cultivable soils have a historical record of annual application of inorganic P compounds, such as potassium dihydrogen phosphate (KH_2_PO_4_) or diammonium phosphate (NH_4_)_2_PO_4_.

Soil samples were collected from seasonal corn cultivated land and reclaimed land after a 30-year period. Four quadrats, each measuring 40 m × 40 m, were established within the experimental area. Soil samples were collected at depths of 0–20 cm, 20–40 cm, 40–60 cm, 60–80 cm, and 80–100 cm using a five-point random sampling method. The study collected a total of 200 samples from 40 sampling locations. Jinhe Farm permitted the work, field site access, and soil sampling in cultivated areas.

The samples were air-dried, gently crushed, and subjected to analysis using a 2 mm sieve. The forms of P were determined using the Hedley extraction method [48], while organic carbon (Corg) and total C were analyzed using a high frequency infrared carbon and sulfur analyzer. pH value was measured through the potentiometric method, total P was quantified via ICP-OES, available P was assessed using the ammonium chloride-hydrochloric acid extraction of molybdenum-antimony colorimetric method, and cation exchange capacity (CEC) was determined employing the Kjeldahl nitrogen analyzer. The fundamental soil characteristics were presented in **Table 1**.

**Table 1:** Basic characteristics of soil in the study area (0–100 cm).

Soil layers (cm)	pH	Total C (%)	Corg (%)	Total N (g kg^-1^)	CEC (mg kg^-1^)	Total P (mg kg^-1^)	Rapidly available K(mg kg^-1^)
0–20	5.87±0.11 (5.78–5.93)	2.28±0.41 (2.06–2.63)	1.82±0.54 (1.49–2.25)	1.70±0.24 (1.51–1.85)	17.1±3.2 (14.9–19.4)	808±83 (759–877)	112±16.7 (99.5–123)
20–40	5.83±0.18 (5.73–5.98)	0.88±0.15b (0.76–0.96)	0.78±0.24 (0.63–0.97)	0.87±0.23 (0.70–1.03)	26.2±2.5 (24.0–27.6)	547±70 (488–587)	158±31 (137–181)
40–60	6.27±0.08 (6.23–6.35)	0.60±0.24 (0.47–0.81)	0.59±0.18 (0.46–0.72)	0.68±0.11 (0.62–0.78)	26.8±1.7 (25.5–27.9)	500±62 (468–556)	168±26 (142–178)
60–80	6.54±0.28 (6.26–6.65)	0.50±0.19 (0.40–0.66)	0.45±0.19 (0.35–0.61)	0.54±0.13 (0.46–0.64)	25.3±1.6 (24.0–26.3)	480±27 (462–500)	170±35 (139–188)
80–100	6.78±0.25 (6.62–6.97)	0.44±0.18 (0.33–0.59)	0.39±0.20 (0.25–0.54)	0.52±0.08 (0.45–0.57)	26.6±2.6 (24.0–27.7)	514±72 (483–585)	196±39 (178–233)

Note: The values enclosed in parentheses represent the range of concentration.

### Phosphate adsorption and desorption

2.50 g of soil was added to each polypropylene copolymer centrifuge tube for precise weighing, followed by the addition of 50 mL of KH_2_PO_4_-NaCl solution with varying concentrations (0, 10, 20, 40, 60, 80, and 100 mg L^-1^) at a pH value of 7. Five drops of chloroform were added to each sample as a microbial activity inhibitor. The tube was subjected to oscillation at a frequency of 150 cycles per minute at a controlled temperature of 25±1°C for a duration of 24 hours in order to attain equilibrium, followed by centrifugation at a speed of 5000 rpm for a period of 10 minutes. The contents of each tube were filtered through a 0.45 μm membrane filter, and the concentration of P in the solution was determined using ascorbate-molybdate phosphate blue colorimetry. The discrepancy between the initial concentration and the equilibrium solution concentration represents the soil’s adsorption capacity. Following the completion of the adsorption experiment, the supernatant was decanted and the residual soil sample was rinsed with 15 mL of saturated NaCl solution. The process was repeated three times to ensure the complete removal of free P. Subsequently, a 50 mL solution of 0.01 mol L^-1^ NaCl (pH = 7) was added, followed by the addition of 5 drops of chloroform to each tube. The tubes were then gently shaken at a temperature of 25°C for a duration of 24 hours. Afterward, centrifugation was performed for a period of 10 minutes, and the resulting P desorption was quantified.

### Statistical analyses

The adsorption process was characterized by employing the Langmuir equation [49]: Q_e_ = kQ_max_C_e_(1+kC_e_) and the Freundlich equation [50, 51]: Q_e_ = aC_e_^1/n^, where Q_e_ (mg kg^−1^) represents the equilibrium P adsorption capacity of soil at a given P concentration C_e_ (mg L^−1^), Q_max_ (mg kg^−1^) denotes the maximum P adsorption capacity of soil, and k (L mg^−1^) signifies the binding strength constant for P at the adsorption site. Furthermore, k·Q_max_ is defined as the maximum buffer capacity for phosphorus adsorption (MBC, L kg^−1^).

The desorption process can be mathematically described by the Langmuir equation: D_e_ = kD_max_C_e_(1+kC_e_) and the Freundlich equation: De = aC_e_^1/n^, where D_e_ (mg kg^−1^) represents the equilibrium amount of P desorbed from the soil at concentration C_e_ (mg L^−1^), k (L mg^−1^) is a constant indicating desorption intensity, and D_max_ (mg kg^−1^) denotes the maximum P desorption capacity. The desorption ratio (D_r_) is defined as the ratio of D_max_ to Q_max_.

## Results and discussion

### Content of different P forms in soil

The bioavailability of soil P varies depending on its form, with available P being the most easily absorbed and utilized by plants in soil. This includes water-soluble P and non-obligate adsorbed P. The P activation coefficient (PAC) represents the ratio of available P to total P, serving as an indicator for assessing the conversion difficulty between these two forms of P. PAC serves as a crucial indicator of soil fertility, with higher values indicating greater potential for promoting plant growth through increased P supply [36].

The water-soluble form of P (H_2_O-P_i_) represents the most efficacious inorganic source of P for plants. The extraction of P from sodium bicarbonate comprises inorganic P (NaHCO_3_-P_i_) and organoP (NaHCO_3_-P_o_) adsorbed onto the surface of polycrystalline P compounds, sesquioxides, or carbonates. The active P is derived from the extraction of H_2_O-P_i_ and NaHCO_3_-P. Inorganic P (NaOH-P_i_) and organic P (NaOH-Po), extracted using sodium hydroxide, are associated with amorphous crystalline aluminum, iron phosphate, and phosphorous bound to humic acid and furic acid. These compounds can be classified as medium active P. The predominant form of P extracted from HCl is calcium-bound P (HCl-P_i_), which exhibits lower efficacy on plants. Residual P (Res-P) refers to inorganic P that is sequestered within sesquioxide, forming a closed-storage system [52].

The available P exhibited variations within the 0–100 cm soil layer, with the highest concentration observed in the uppermost 0–20 cm and the lowest concentration found in the deeper 60–80 cm. The available P content exhibited an initial increase followed by a subsequent decrease with increasing soil depth (**Table 2**). Firstly, the enrichment of organic matter and active microbial processes promoted efficient nutrient transformation in the soil, resulting in increased accessibility of P and noticeable formation of surface-bound P compounds. Conversely, as a consequence of plant roots predominantly occupying the 20–80 cm depth range within the soil profile, their physiological activities and nutrient uptake significantly diminished the concentration of available P in the soil. Additionally, the dense texture of the underlying soil effectively impeded P migration within the soil profile, thereby confirming the relatively high P concentration observed at depths ranging from 80 to 100 cm. This finding further supports the presence of a characteristic cohesive layer in black soils. PAC and available P exhibited a consistent pattern, as shown in **Table 2**. The highest PAC value was observed in the 0–20 cm soil layer, indicating enhanced availability of P in the soil. Furthermore, all layers displayed a PAC exceeding 2.0%, suggesting a high utilization rate of black soil P.

**Table 2:** Results of different P forms in soil (mg kg^-1^).

Soil layer	0–20 cm	20–40 cm	40–60 cm	60–80 cm	80–100 cm
Available P	67.7±13.0a	25.3±8.6b	19.8±13.8b	14.0±1.1b	21.7±6.7b
H_2_O- P_i_	11.7±1.7a	6.9±2.5b	5.2±4.0b	3.0±1.2b	4.1±3.3b
NaHCO_3_-P_i_	45.9±11.2a	14.1±4.8b	13.6±13.1b	8.3±0.2b	13.7±3.2b
NaHCO_3_-P_o_	88.9±37.7a	40.1±6.9b	36.5±6.9b	26.8±0.3b	31.7±4.5b
NaOH-P_i_	120±4.6a	75.6±8.0b	65.6±12.8bc	59.3±17.2bc	54.1±33.5c
NaOH-P_o_	308±16.1a	221±19.7b	211±4.3b	223±31.7b	207±36.0b
HCl-P	80.2±6.2a	49.8±26.2b	36.2±12.7b	27.7±12.7b	47.6±28.2b
Res-P	139±11.6a	125±10.7bc	118±2.2bc	120±11.9bc	114±27.6c
PAC (%)	8.3±0.5a	4.6±0.8b	4.0±2.2b	2.9±0.4b	4.3±1.7b

Note: Different letters within a line are significantly different at P < 0.05.

The concentrations of active P species, including H_2_O-P_i_, NaHCO_3_-P_i_, and NaHCO_3_-P_o_ in soil profiles were found to be relatively low. Among these species, the content of H_2_O-P_i_, which is considered most beneficial for plant growth, exhibited the lowest concentration ranging from 0.64% to 1.47% of the total P content in the soil. The content of medium active P, NaOH-P, is significantly high, with NaOH-P_o_ being the predominant form at 38.8–47.6%. This finding highlights the crucial role of black soil as a potential reservoir for P and aligns with the consensus among numerous scholars [16, 53].

### Adsorption characteristics

#### P adsorption isotherms

**Fig 1:**
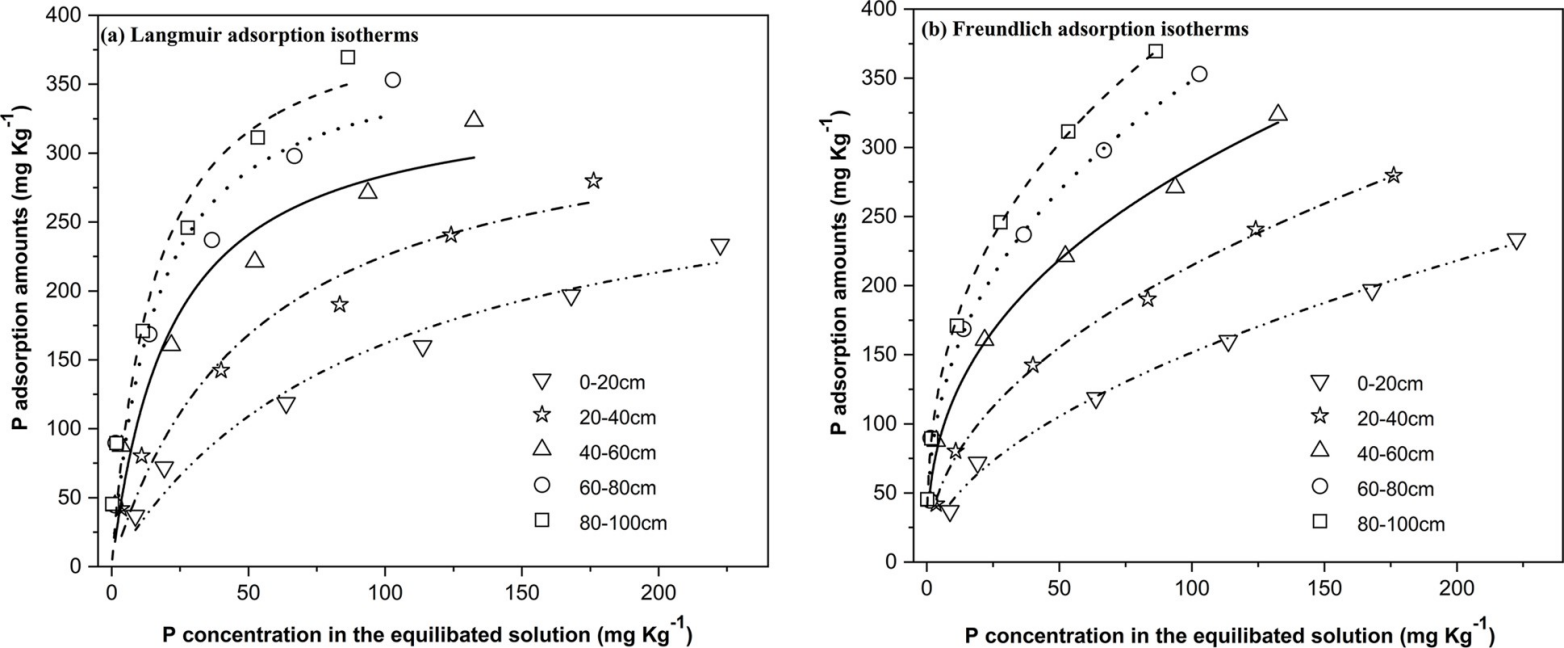
Isotherms of soil P adsorption in vertical spatial distribution.

The amount of P adsorbed by black soil at different depths increases with the concentration of the P equilibrium solution, as illustrated in **Fig 1**. When the concentration of P in the equilibrium solution is low, a steeper slope can be observed in the curve depicting the relationship between adsorption capacity and concentration of the equilibrium solution, indicating a higher affinity for soil adsorption. The increase in P concentration in the equilibrium solution leads to a deceleration of the adsorption capacity, resulting in a smaller slope of the curve and a decrease in soil’s adsorption capacity. The adsorption process of P can be categorized into chemical and physical processes [49]. At low P concentration, chemisorption dominates the adsorption process, leading to its rapid completion. Ion exchange and ligand exchange are likely to be the primary mechanisms contributing to the high rate of adsorption [46]. The P in the liquid undergoes physical adsorption and gradually attaches to the soil, a process referred to as the slow adsorption stage.

The Langmuir equation and Freundlich equation were employed to fit the isothermal adsorption data of soil P in different layers of black soil. The resulting correlation coefficients ranged from 0.906 to 0.962 and from 0.986 to 0.994, respectively, indicating a high level of significance (**Table 3**). The P adsorption characteristics of soil can be described by either of the two equations, which is consistent with findings from previous studies. The determination of P availability in soil and its adsorption capacity are commonly assessed through the Q_max_ and MBC calculated using the Langmuir adsorption isotherm [46].

**Table 3:** Adsorption characteristics of P in different soil depths.

Soil layers (cm)	Langmuir equation		Freundlich equation		Q_max_ (mg kg^-1^)	MBC (L kg^-1^)	DPS (%)
	Q_e_ = kQ_max_C_e_(1+kC_e_)	R^2^	Q_e_ = aC_e_^1/n^	R^2^			
0–20	Q_e_ = 3.1C_e_/(1+0.01C_e_)	0.962^**^	Q_e_ = 13.6C_e_^0.52^	0.994^**^	313.8±17.1a	3.1±0.14a	21.6±2.4a
20–40	Q_e_ = 6.8C_e_/(1+0.02C_e_)	0.954^**^	Q_e_ = 25.0C_e_^0.47^	0.991^**^	345.9±27.1b	6.8±0.17b	7.4±1.4b
40–60	Q_e_ = 17.3C_e_/(1+0.05C_e_)	0.921^**^	Q_e_ = 62.3C_e_^0.37^	0.986^**^	352.4±20.0b	17.3±0.38c	5.6±0.8b
60–80	Q_e_ = 22.7C_e_/(1+0.06C_e_)	0.906^**^	Q_e_ = 72.9C_e_^0.36^	0.989^**^	381.6±31.2c	22.7±0.88d	3.8±0.6b
80–100	Q_e_ = 28.8C_e_/(1+0.07C_e_)	0.925^**^	Q_e_ = 48.6C_e_^0.38^	0.990^**^	411.9±20.6d	28.8±0.73e	5.3±1.0b

Note: Different letters within a column are significantly different at P < 0.05. The DPS is defined as the ratio of the concentration of available P to Q_max_.

#### P adsorption parameters

The parameter Q_max_ represents the P adsorption capacity of soil, indicating the number of P adsorption sites per unit weight of soil. It is extensively employed for evaluating the soil’s ability to adsorb P. The Q_max_ of the black soil in the vertical space, as presented in **Table 3**, exceeds 313.8 mg kg^-1^ and exhibits a gradual increase with soil depth ranging from 0 to 100 cm. It reaches its maximum value within the range of 80–100 cm, where Q_max_ reaches 411.9 mg kg^-1^. The conclusion aligns with the findings of Amarh et al. [54]. The results indicated that the 0–20 cm soil layer exhibited limited P fixation capacity, whereas the 80–100 cm soil layer harbored a substantial reservoir of P.

The MBC, which is a comprehensive parameter of Q_max_ and k [49], positively correlates with the adsorption capacity of P [55]. The results presented in **Table 3** demonstrate a positive correlation between soil depth and MBC in the vertical space. Notably, the highest MBC was observed in the 80-100cm soil layer, indicating a substantial P storage capacity within the soil and limited replenishment ability of P in the soil solution.

The degree of P adsorption saturation (DPS) can serve as a reliable indicator to assess both the capacity and intensity of soil P supply, thus playing a crucial role in evaluating soil P availability. The findings of this study revealed that the DPS in black soil ranged from 3.8% to 21.6%. In contrast to the variation pattern observed for Q_max_, the DPS levels decreased with increasing depth across different soil layers. Notably, the highest DPS was observed in the 0–20 cm soil layer, indicating a relatively low P adsorption capacity of the soil.

### Desorption characteristics

#### P desorption isotherms

**Fig 2:**
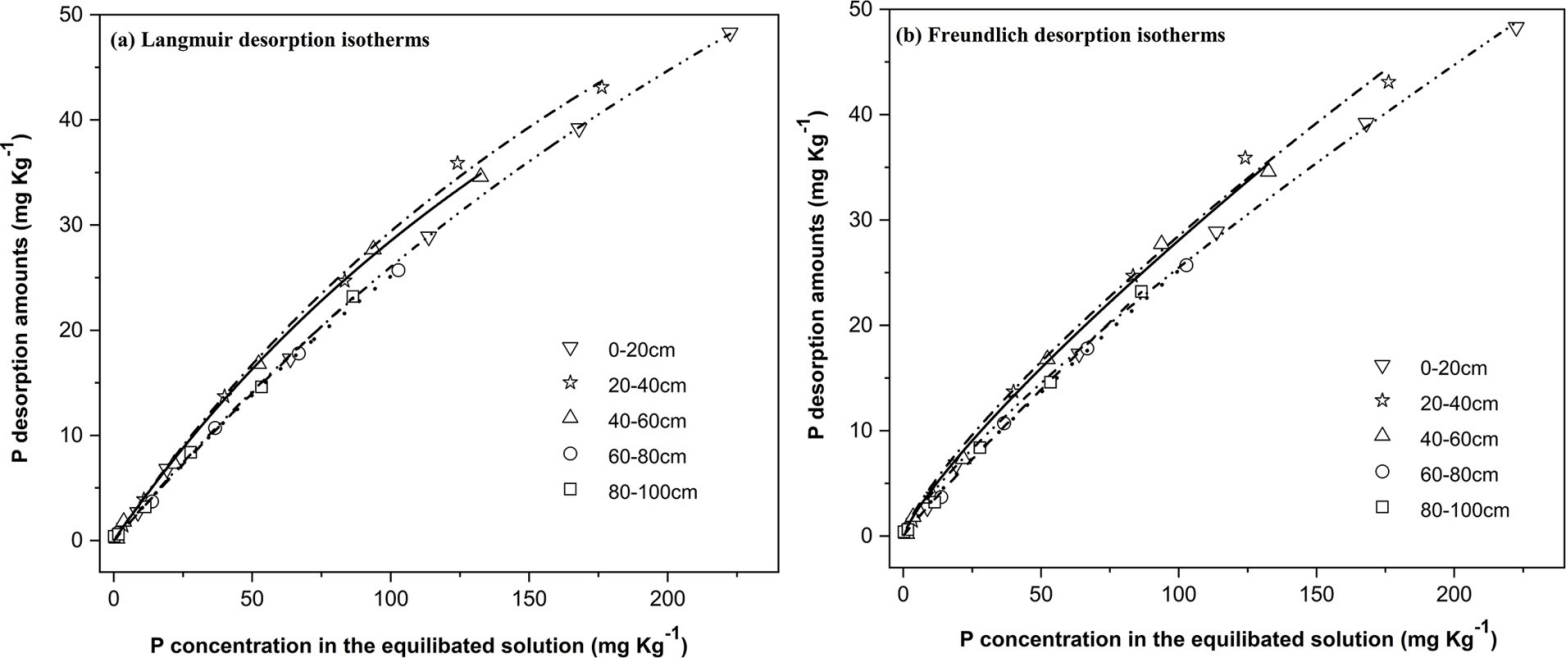
Isotherms of soil P desorption in vertical spatial distribution.

The process of desorption, as the reverse reaction of soil P adsorption, is considered to be more significant than adsorption itself. This process involves the recycling of adsorbed P and has a profound impact on the availability of soil P. The Langmuir equation and Freundlich equation were employed to fit the isothermal desorption data of soil P in different layers of black soil. The correlation coefficients ranged from 0.991 to 0.997 and 0.990 to 0.995, respectively, indicating highly significant associations (**Table 4**). The results depicted in **Fig 2** demonstrate that a substantial quantity of P adsorbed by the soil can undergo desorption to a certain extent, subsequently being released back into the solution. The maximum P desorption capacity (D_max_), desorption ratio (D_r_), and readily desorbable P in soil (RDP) were employed as indicators to assess the soil’s ability to release P [56].

**Table 4:** Desorption characteristics of P in different soil depths.

Soil layers (cm)	Langmuir equation		Freundlich equation		D_max_ (mg kg^-1^)	D_r_ (%)	RDP (mg kg^-1^)
	D_e_ = kD_max_C_e_(1+kC_e_)	R^2^	D_e_ = aC_e_^1/n^	R^2^			
0–20	D_e_ = 0.32C_e_(1+0.002C_e_)	0.991^**^	D_e_ = 0.61C_e_^0.81^	0.990^**^	157.7±2.4d	50.4±3.0b	3.35±0.67c
20–40	D_e_ = 0.36C_e_(1+0.003C_e_)	0.997^**^	D_e_ = 0.77C_e_^0.78^	0.992^**^	120.7±1.6b	35.1±2.7a	2.05±0.57b
40–60	D_e_ = 0.34C_e_(1+0.003C_e_)	0.992^**^	D_e_ = 0.65C_e_^0.82^	0.995^**^	112.8±5.8a	32.1±2.7a	1.02±0.28a
60–80	D_e_ = 0.27C_e_(1+0.002C_e_)	0.993^**^	D_e_ = 0.42C_e_^0.89^	0.992^**^	135.5±3.1c	35.7±2.4a	1.10±0.28a
80–100	D_e_ = 0.22C_e_(1+0.001C_e_)	0.991^**^	D_e_ = 0.36C_e_^0.93^	0.994^**^	215.7±12.8e	52.5±5.1b	1.43±0.42a

Note: Different letters within a column are significantly different at P < 0.05.

#### Desorption parameters

The D_max_ values for the black soil ranged from 112.8 mg kg^-1^ to 215.7 mg kg^-1^ within the 0–100 cm soil layer. In terms of vertical distribution, the desorption of soil P exhibited an initial decrease followed by an increase with increasing depth. The desorption of soil P was found to be the lowest in the 40–60 cm soil layer, while it exhibited the highest levels in the 80–100 cm soil layer.

The D_r_ of soil P can serve as an indicator of the soil colloid’s capacity for releasing P. Due to variations in P supply intensity and capacity across different soil layers, the degree of soil P desorption varies accordingly. As soil depth increases, the rate of P desorption initially decreases before increasing again, with the highest rate observed at a depth of 80–100 cm.

The results presented in **Table 4** demonstrated that the RDP content in the 0–100 cm soil layer ranges from 1.02 mg kg^-1^ to 3.35 mg kg^-1^, displaying a decreasing trend followed by an increasing trend with progressive soil depth vertically. The results suggest that the 0–20 cm soil layer exhibits a pronounced capacity for P release into the soil environment. However, there was a relatively limited availability of absorbed and utilized P within the 40–60 cm soil layer.

## Discussion

The variation of soil properties, including organic matter content, clay composition, pH value, as well as the presence of Fe and Al oxides across different layers of black soil, exerts an influence on the availability and adsorption-desorption of soil P [57–60]. Soil organic matter serves as a crucial reservoir of P, and alterations in its content can lead to variations in soil P constituents [53]. The correlation analysis (**Table 5**) revealed a significant positive relationship between the contents of total P, available P, H_2_O-P_i_, Na_2_CO_3_-P_i_, Na_2_CO_3_-P_0_, NaOH-P_i_, NaOH-P_t_ and Res-P in soil profiles with the organic matter content. This suggests that the distribution of P content in black soil profiles is influenced by the organic matter content [61].

**Table 5:** Pearson’s correlation coefficient between P adsorption-desorption parameters, P morphology, Corg, pH and CEC.

Index	Corg	pH	CEC	Total P	available P	H_2_O-P_i_	Na_2_CO_3_-P_i_	Na_2_CO_3_-P_o_	NaOH-P_i_	NaOH-P_o_	HCl-P	Q_max_	MBC	DPS	D_max_	D_r_	RDP
Corg	1.00	-0.03	0.11	0.77^**^	0.47^**^	0.60^**^	0.44^**^	0.57^**^	0.67^**^	0.61^**^	0.17	-0.78^**^	-0.74^**^	0.60^**^	0.15	0.66^**^	0.70^**^
pH		1.00	-0.12	0.096	-0.04	−	-0.07	-0.09	-0.03	−	−	0.072	-0.08	-0.14	-0.18	-0.13	-0.07
CEC			1.00	−	-0.06	-0.13	-0.03	-0.04	0.07	−	-0.30^**^	-0.11	0.11	−	0.16	0.19	−
Total P				1.00	0.29^**^	0.45^**^	0.26^**^	0.28^**^	0.85^**^	0.77^**^	0.30^**^	-0.78^**^	-0.62^**^	0.42^**^	−	0.55^**^	0.47^**^
available P					1.00	0.84^**^	0.99^**^	0.87^**^	0.25^*^	0.23^*^	0.28^**^	-0.21^*^	-0.40^**^	0.98^**^	0.13	0.31^**^	0.54^**^
H_2_O-P_i_						1.00	0.76^**^	0.74^**^	0.34^**^	0.25^*^	0.43^**^	-0.34^**^	-0.52^**^	0.85^**^	−	0.31^**^	0.52^**^
Na_2_CO_3_-P_i_							1.00	0.88^**^	0.25^*^	0.21	0.24^*^	-0.19	-0.37^**^	0.96^**^	0.16	0.31^**^	0.54^**^
Na_2_CO_3_-P_o_								1.00	0.26^*^	0.16	0.27^*^	-0.25^*^	-0.49^**^	0.86^**^	0.11	0.33^**^	0.67^**^
NaOH-P_i_									1.00	0.76^**^	0.23^*^	-0.73^**^	-0.51^**^	0.36^**^	0.06	0.51^**^	0.35^**^
NaOH-P_o_										1.00	−	-0.67^**^	-0.52^**^	0.33^**^	−	0.46^**^	0.40^**^
HCl-P											1.00	-0.09	-0.13	0.27^*^	−	0.09	0.12
Res-P												-0.56^**^	-0.36^**^	0.19	0.16	0.44^**^	0.31^**^
Q_max_												1.00	0.78^**^	-0.37^**^	−	-0.59^**^	-0.47^**^
MBC													1.00	-0.50^**^	0.09	-0.42^**^	-0.62^**^
DPS														1.00	0.15	0.42^**^	0.61^**^
D_max_															1.00	0.79^**^	0.36^**^
D_r_																1.00	0.65^**^
RDP																	1.00

**Significant at P < 0.01

*Significant at P < 0.05.

The soil organic matter exhibits a significant negative correlation with both Q_max_ and the MBC of soil P, while demonstrating a significant positive correlation with DPS, D_r_, and RDP. The levels of available P, H_2_O-P_i_, Na_2_CO_3_-P_i_, and Na_2_CO_3_-P_o_ exhibited significant positive correlations with DPS and RDP, while displaying a significant negative correlation with MBC. NaOH-P_i_ and NaOH-P_o_ were positively correlated with D_r_, but negatively correlated with Q_max_ and MBC (**Table 5**). The conclusion aligns with the findings of Yang et al. [62]. The black soil surface exhibits a substantial organic matter content, whereby the decomposition process generates organic acids that facilitate the dissolution of insoluble phosphate. Additionally, corresponding organic anions compete for soil adsorption sites, thereby enhancing P availability and mitigating its fixation by the soil [57, 63]. Simultaneously, surface soil organic matter, particularly humic acid humus, can form complexes with inorganic substances such as clay minerals, iron oxide, aluminum, and calcium carbonate [43, 64, 65]. This process effectively diminishes the physical and chemical adsorption potential of soil mineral colloid for P, facilitating the desorption of P from the soil surface into the soil solution [56, 66]. This explains why the contents of available P, H_2_O-P_i_, Na_2_CO_3_-P_i_, and Na_2_CO_3_-P_o_ are highest in the 0–20 cm soil layer. Consequently, the adsorption capacity, Q_max_, and MBC of the 0–20 cm soil layer in the soil profile were significantly lower compared to other soil layers. Conversely, D_r_ and RDP content exhibited higher values.

With increasing soil depth, the organic matter content in soil exhibited a decline, accompanied by a reduction in the competitive adsorption capacity of organic anions [67]. Simultaneously, as soil depth increased, all forms of phosphorus exhibited decreased contents, particularly NaOH-P_i_ and NaOH-P_o_. Although soil porosity decreased with increasing soil depth (S1 Table), which was not conducive to phosphorus adsorption, the results showed an increase in both the Q_max_, and MBC of the soil. This indicates that the impact of soil porosity on phosphorus adsorption is not significant in this study.

The clay content exhibited an initial decrease followed by an increase with the progressive increment of soil depth (S1 Table). The presence of the clay layer within the depth range of 80–100 cm in black soil leads to an increase in clay content. Consequently, the soil clay forms an organic-inorganic complex with the anionic groups present in the soil layer, thereby shielding the adsorption sites and reducing P adsorption in the soil. This phenomenon enhances P desorption, resulting in a relatively high Dr and RDP conten.

### Implications for sustainable soil fertility management

The adsorption capacity, maximum adsorption capacity, and maximum adsorption buffer capacity of the 0–20 cm soil layer in the soil profile were significantly lower compared to other soil layers, while exhibiting higher desorption ratio and easily desorbed phosphorus content. However, as soil depth increases, there is a decrease in soil organic matter content and the competitive adsorption ability of organic anions. Conversely, there is an increase in soil phosphorus adsorption capacity, maximum adsorption capacity, maximum adsorption buffer capacity, and a decrease in phosphorus desorption ratio. The soil serves as a transient reservoir for phosphorus nutrients, and its capacity for adsorption and desorption not only constrains the efficacy of phosphorus fertilizers but also influences the loss of soil phosphorus.

The presence of organic matter in soil can enhance the phosphorus content, thereby exerting a substantial influence on crop growth and yield. Nevertheless, the topsoil in this region exhibits an elevated range of available phosphorus (>20 ppm), potentially attributed to frequent utilization of mineralized phosphorus. The findings of this study demonstrate that the organic matter content in the topsoil (0-20cm) ranges from 1.49% to 2.25%, accompanied by Q_max_, MBC, and DPS values of 313.8 mg kg^-1^, 3.1 L kg^-1^, and 21.6%, respectively. The outcomes derived from this investigation hold practical significance for managing soil fertility, regulating phosphorus adsorption and desorption processes within soils, as well as controlling crop phosphorus nutrition.

## Conclusion

The vertical spatial variation characteristics of soil adsorption and desorption in the black soil area of northeast China were investigated based on the determination and analysis of key parameters including Q_max_, MBC, DPS, D_max_, D_r_ and RDP in the 0–100 cm soil layer. The results demonstrated a highly significant fitting degree (p< 0.01) between the isothermal adsorption and desorption data of P in different soil layers and both the Langmuir equation and Freundlich equation. The vertical spatial variation of Q_max_ showed a positive correlation with soil depth, while the DPS of P exhibited an inverse trend, with a significant difference observed among different levels (p<0.05). The concentrations of D_r_ and RDP in the 0–20 cm soil layer within the study area exhibited the highest values, indicating a substantial phosphorus supply capacity at this specific depth range. The P availability exhibited a certain extent of decrease with increasing soil depth. The soil organic matter exhibits a significant negative correlation with both Q_max_ and the MBC of soil P, while demonstrating a significant positive correlation with DPS, D_r_, and RDP. The findings of this study offer theoretical underpinning for the management of soil fertility and regulation of phosphorus nutrition in crops.

## Supporting information

S1 Fig Vertical distribution characteristics of different phosphorus forms in the study area. (TIF)

S2 Fig Vertical distribution characteristics of soil available phosphorus, inorganic phosphorus and organic phosphorus in the study area. (TIF)

S3 Fig Principal component analysis of soil physicochemical indices, organic carbon, and phosphorus forms in the vertical dimension. (TIF)

S4 Fig Principal component analysis of adsorption and desorption parameters for organic carbon and soil P in the vertical dimension. (TIF)

S1 Table Mechanical composition of soil in the study area (0–100 cm). (DOC)

## References

[1] Rosengren U, Göransson H, Jönsson U, Stjernquist I, Thelin G, Wallander H. Functional Biodiversity Aspects on the Nutrient Sustainability in Forests-Importance of Root Distribution. Journal of Sustainable Forestry. 2006;21(2–3):77–100. 10.1300/J091v21n02_06.

[2] Yang X, Kong Y, Guo E, Chen X, Li L. Organic acid regulation of inorganic phosphorus release from Mollisols with different organic matter contents. Soil Use and Management. 2021;38(1):576–83. 10.1111/sum.12710.

[3] Zhu H, Bing H, Wu Y, Sun H, Zhou J. Low molecular weight organic acids regulate soil phosphorus availability in the soils of subalpine forests, eastern Tibetan Plateau. Catena. 2021;203. 10.1016/j.catena.2021.105328.

[4] Tiruneh GA, Meshesha DT, Adgo E, Tsunekawa A, Haregeweyn N, Fenta AA, et al. Use of soil spectral reflectance to estimate texture and fertility affected by land management practices in Ethiopian tropical highland. Plos One. 2022;17(7):e0270629. doi: 10.1371/journal.pone.0270629 35862343 PMC9302783

[5] Liu G, Chen L, Jiang Z, Zheng H, Dai Y, Luo X, et al. Aging impacts of low molecular weight organic acids (LMWOAs) on furfural production residue-derived biochars: Porosity, functional properties, and inorganic minerals. Science of The Total Environment. 2017;607–608:1428–36. 10.1016/j.scitotenv.2017.07.046. 28746993

[6] Tiecher T, Gomes MV, Ambrosini VG, Amorim MB, Bayer C. Assessing linkage between soil phosphorus forms in contrasting tillage systems by path analysis. Soil and Tillage Research. 2018;175:276–80. 10.1016/j.still.2017.09.015.

[7] Abdala DB, Ghosh AK, da Silva IR, de Novais RF, Alvarez Venegas VH. Phosphorus saturation of a tropical soil and related P leaching caused by poultry litter addition. Agriculture, Ecosystems & Environment. 2012;162:15–23. 10.1016/j.agee.2012.08.004.

[8] Tiruneh GA, Alemayehu TY, Meshesha DT, Vogelmann ES, Reichert JM, Haregeweyn N. Spatial variability of soil chemical properties under different land-uses in Northwest Ethiopia. Plos one. 2021;16(6):e0253156. doi: 10.1371/journal.pone.0253156 34161393 PMC8241222

[9] Oral A, Uygur V. Effects of low-molecular-mass organic acids on P nutrition and some plant properties of Hordeum vulgare. Journal of Plant Nutrition. 2018;41(11):1482–90. 10.1080/01904167.2018.1458866.

[10] Santos SR, Silva EdB, Alleoni LRF, Grazziotti PH. Citric acid influence on soil phosphorus availability. Journal of Plant Nutrition. 2017;40(15):2138–45. 10.1080/01904167.2016.1270312.

[11] Zhu J, Li M, Whelan M. Phosphorus activators contribute to legacy phosphorus availability in agricultural soils: A review. Science of The Total Environment. 2018;612:522–37. doi: 10.1016/j.scitotenv.2017.08.095 28865270

[12] Schoumans OF, Chardon WJ. Phosphate saturation degree and accumulation of phosphate in various soil types in The Netherlands. Geoderma. 2015;237–238:325–35. 10.1016/j.geoderma.2014.08.015.

[13] Rheinheimer DdS, Fornari MR, Bastos MC, Fernandes G, Santanna MA, Calegari A, et al. Phosphorus distribution after three decades of different soil management and cover crops in subtropical region. Soil and Tillage Research. 2019;192:33–41. 10.1016/j.still.2019.04.018.

[14] Su N, Xie G, Mao Z, Li Q, Chang T, Zhang Y, et al. The effectiveness of eight-years phosphorus reducing inputs on double cropping paddy: Insights into productivity and soil-plant phosphorus trade-off. Science of The Total Environment. 2023;866:161429. doi: 10.1016/j.scitotenv.2023.161429 36623670

[15] Zhang S, Huffman T, Zhang X, Liu W, Liu Z. Spatial distribution of soil nutrient at depth in black soil of Northeast China: a case study of soil available phosphorus and total phosphorus. Journal of Soils and Sediments. 2014;14(11):1775–89. 10.1007/s11368-014-0935-z.

[16] Yang X, Chen X, Guo E, Yang X. Path analysis of phosphorus activation capacity as induced by low-molecular-weight organic acids in a black soil of Northeast China. Journal of Soils and Sediments. 2018;19(2):840–7. 10.1007/s11368-018-2034-z.

[17] Chen X, Yan X, Wang M, Cai Y, Weng X, Su D, et al. Long-term excessive phosphorus fertilization alters soil phosphorus fractions in the acidic soil of pomelo orchards. Soil and Tillage Research. 2022;215:105214. 10.1016/j.still.2021.105214.

[18] Wang YT, Zhang TQ, O’Halloran IP, Tan CS, Hu QC. A phosphorus sorption index and its use to estimate leaching of dissolved phosphorus from agricultural soils in Ontario. Geoderma. 2016;274:79–87. 10.1016/j.geoderma.2016.04.002.

[19] Zhang Z-S, Song X-L, Lu X-G, Xue Z-S. Ecological stoichiometry of carbon, nitrogen, and phosphorus in estuarine wetland soils: influences of vegetation coverage, plant communities, geomorphology, and seawalls. Journal of Soils and Sediments. 2013;13:1043–51. 10.1007/s11368-013-0693-3.

[20] Zhu M, Guo Y, Cao X, Yang Y, Du Q, Lou J, et al. Shelterbelt-farmland differences in P fractions interacted with soil alkalization, geoclimatic conditions, and soil fungi in Northeast China Plain. Journal of Soils and Sediments. 2023;23(11):3937–57. 10.1007/s11368-023-03551-6.

[21] Zhu M, Cheng G, Zhang X, Guo Y, Wu Y, Wang Q, et al. Shelterbelts increased soil inorganic carbon but decreased nitrate nitrogen, total phosphorus, and bulk density relative to neighbor farmlands depending on tree growth, geoclimate, and soil microbes in the Northeast China Plain. Catena. 2023;231:107344. 10.1016/j.catena.2023.107344.

[22] Tian H, Li Z, Song Z, Han H, Cheng X. Structural Equation Modeling of Phosphorus Transformations in Soils of Larix principis-rupprechtii Mayr. Plantations. Forests. 2023;14(9):1811. 10.3390/f14091811.

[23] Cao X, Ji Q, Wei C, Xiao L, Zhang P, Mao R, et al. Plant succession and geo-topography determined forest soil P and nine fraction-Ps across a larch forest chronosequence in the northmost region of China. Plant and Soil. 2023;486(1):681–703. 10.1007/s11104-023-05900-3.

[24] Hu X, Gu H, Liu J, Wei D, Zhu P, Cui Xa, et al. Metagenomic strategies uncover the soil bioavailable phosphorus improved by organic fertilization in Mollisols. Agriculture, Ecosystems & Environment. 2023;349:108462. 10.1016/j.agee.2023.108462.

[25] Cui H, Fan M, Wang Y, Zhang X, Xu W, Li Y, et al. Impacts of mowing and N addition on soil organic phosphorus mineralization rates in a semi-natural grassland in Northeast China. Plant and Soil. 2023;482(1):7–23. 10.1007/s11104-022-05670-4.

[26] Qin L, Jiang M, Freeman C, Zou Y, Gao C, Tian W, et al. Agricultural land use regulates the fate of soil phosphorus fractions following the reclamation of wetlands. Science of The Total Environment. 2023;863:160891. doi: 10.1016/j.scitotenv.2022.160891 36526180

[27] Zhao Y, Hao Y, Cheng K, Wang L, Dong W, Liu Z, et al. Artificial humic acid mediated migration of phosphorus in soil: Experiment and modelling. CATENA. 2024;238:107896. 10.1016/j.catena.2024.107896.

[28] Yan S, Liu C, Li J, Li J, Cui C, Fan J, et al. Changes in the soil phosphorus supply with rice straw return in cold region. Agronomy. 2023;13(9):2214. 10.3390/agronomy13092214.

[29] Yang X, He P, Zhang Z, You M, Wu X, Li L-J. Straw return, rather than warming, alleviates microbial phosphorus limitation in a cultivated Mollisol. Applied Soil Ecology. 2023;186:104821. 10.1016/j.apsoil.2023.104821.

[30] Zhang S, Wang W, Guo M, Wang H, Gao L, Shen Q, et al. Spatial heterogeneity of soil available phosphorus changed after freeze and thaw cycles in Mollisols of a watershed. Nutrient Cycling in Agroecosystems. 2023;127(1):101–17. 10.1007/s10705-023-10307-8.

[31] Li G, Zhao H, Wang G, Cong J, Ji S, Gao C. Response of phosphorus distribution to burn frequency and seasonality in the Sanjiang Plain wetlands (Northeast China). Ecological Indicators. 2023;154:110673. 10.1016/j.ecolind.2023.110673.

[32] Song Z, Li Z, Luo Y, Liu Y. Allocation strategies of carbon, nitrogen and phosphorus following a gradient of wildfire severities. Journal of Plant Ecology. 2022;15(2):347–58. 10.1093/jpe/rtab099.

[33] Liu L, Ouyang W, Zhang W, Gao X, He M, Lin C. Future warming-induced phosphorus loss mitigated by land conversion and degradation. Soil and Tillage Research. 2022;224:105526. 10.1016/j.still.2022.105526.

[34] Yang X, Qiu S, Wang C, Hao L. The impact of climate and land use changes on nitrogen and phosphorus pollution in the Luhun Lake Basin, China. Frontiers in Earth Science. 2024;11:1302804. 10.3389/feart.2023.1302804.

[35] Romanyà J, Blanco-Moreno JM, Sans FX. Phosphorus mobilization in low-P arable soils may involve soil organic C depletion. Soil Biology and Biochemistry. 2017;113:250–9. 10.1016/j.soilbio.2017.06.015.

[36] Wu Q, Zhang S, Zhu P, Huang S, Wang B, Zhao L, et al. Characterizing differences in the phosphorus activation coefficient of three typical cropland soils and the influencing factors under long-term fertilization. PLOS ONE. 2017;12(5):e0176437. doi: 10.1371/journal.pone.0176437 28467425 PMC5415111

[37] Malik MA, Marschner P, Khan KS. Addition of organic and inorganic P sources to soil–Effects on P pools and microorganisms. Soil Biology and Biochemistry. 2012;49:106–13. 10.1016/j.soilbio.2012.02.013.

[38] Koch M, Kruse J, Eichler-Löbermann B, Zimmer D, Willbold S, Leinweber P, et al. Phosphorus stocks and speciation in soil profiles of a long-term fertilizer experiment: Evidence from sequential fractionation, P K-edge XANES, and 31P NMR spectroscopy. Geoderma. 2018;316:115–26. 10.1016/j.geoderma.2017.12.003.

[39] Borges BMMN, Abdala DB, Souza MFd, Viglio LM, Coelho MJA, Pavinato PS, et al. Organomineral phosphate fertilizer from sugarcane byproduct and its effects on soil phosphorus availability and sugarcane yield. Geoderma. 2019;339:20–30. 10.1016/j.geoderma.2018.12.036.

[40] Zhou J, Guan D, Zhou B, Zhao B, Ma M, Qin J, et al. Influence of 34-years of fertilization on bacterial communities in an intensively cultivated black soil in northeast China. Soil Biology and Biochemistry. 2015;90:42–51. 10.1016/j.soilbio.2015.07.005.

[41] Chien SH, Prochnow LI, Cantarella H. Chapter 8 Recent Developments of Fertilizer Production and Use to Improve Nutrient Efficiency and Minimize Environmental Impacts. Advances in Agronomy. 102: Academic Press; 2009. p. 267–322. 10.1016/S0065-2113(09)01008-6.

[42] Yang X, Chen X, Yang X. Effect of organic matter on phosphorus adsorption and desorption in a black soil from Northeast China. Soil and Tillage Research. 2019;187:85–91. 10.1016/j.still.2018.11.016.

[43] Gérard F. Clay minerals, iron/aluminum oxides, and their contribution to phosphate sorption in soils-A myth revisited. Geoderma. 2016;262:213–26. 10.1016/j.geoderma.2015.08.036.

[44] Bhattacharyya P, Nayak AK, Shahid M, Tripathi R, Mohanty S, Kumar A, et al. Effects of 42-year long-term fertilizer management on soil phosphorus availability, fractionation, adsorption–desorption isotherm and plant uptake in flooded tropical rice. The Crop Journal. 2015;3(5):387–95. 10.1016/j.cj.2015.03.009.

[45] Debicka M, Kocowicz A, Weber J, Jamroz E. Organic matter effects on phosphorus sorption in sandy soils. Archives of Agronomy and Soil Science. 2016;62(6):840–55. 10.1080/03650340.2015.1083981.

[46] Lai DYF, Lam KC. Phosphorus sorption by sediments in a subtropical constructed wetland receiving stormwater runoff. Ecological Engineering. 2009;35(5):735–43. 10.1016/j.ecoleng.2008.11.009.

[47] Yang Q, Ai X, Sheng M, Ai S, Wang Y, Ai Y. Differences in the distribution, availability, and sorption-desorption isotherms of phosphorus fractions in soil aggregates from cut slopes with different restoration methods. Soil and Tillage Research. 2023;234:105822. 10.1016/j.still.2023.105822.

[48] Hedley MJ, Stewart JWB, Chauhan BS. Changes in Inorganic and Organic Soil Phosphorus Fractions Induced by Cultivation Practices and by Laboratory Incubations. Soil Science Society of America Journal. 1982;46(5):970–6. 10.2136/sssaj1982.03615995004600050017x.

[49] Wang L, Liang T. Effects of exogenous rare earth elements on phosphorus adsorption and desorption in different types of soils. Chemosphere. 2014;103:148–55. doi: 10.1016/j.chemosphere.2013.11.050 24342358

[50] Ye H, Chen F, Sheng Y, Sheng G, Fu J. Adsorption of phosphate from aqueous solution onto modified palygorskites. Separation and Purification Technology. 2006;50(3):283–90. 10.1016/j.seppur.2005.12.004.

[51] Muendo BM, Shikuku VO, Getenga ZM, Lalah JO, Wandiga SO, Rothballer M. Adsorption-desorption and leaching behavior of diuron on selected Kenyan agricultural soils. Heliyon. 2021;7(2):e06073. doi: 10.1016/j.heliyon.2021.e06073 33604468 PMC7875825

[52] Wang Y, Chen X, Whalen JK, Cao Y, Quan Z, Lu C, et al. Kinetics of inorganic and organic phosphorus release influenced by low molecular weight organic acids in calcareous, neutral and acidic soils. Journal of Plant Nutrition and Soil Science. 2015;178(4):555–66. 10.1002/jpln.201500047.

[53] Guo S-L, Dang T-H, Hao M-D. Phosphorus Changes and Sorption Characteristics in a Calcareous Soil Under Long-Term Fertilization1 1Project supported by the National Basic Research Program of China (No. 2005CB121102), the Knowledge Innovation Program of the Chinese Academy of Sciences (No. KZCX2-YW-424-2) and the West Star Foundation of the Chinese Academy of Sciences. Pedosphere. 2008;18(2):248–56. 10.1016/S1002-0160(08)60014-4.

[54] Amarh F, Voegborlo RB, Essuman EK, Agorku ES, Tettey CO, Kortei NK. Effects of soil depth and characteristics on phosphorus adsorption isotherms of different land utilization types: Phosphorus adsorption isotherms of soil. Soil and Tillage Research. 2021;213:105139. 10.1016/j.still.2021.105139.

[55] Chakraborty R, Sharma VK, Das D, Biswas DR, Mahapatra P, Shahi DK, et al. Change in phosphorus availability, fractions, and adsorption-desorption by 46-years of long-term nutrient management in an Alfisol of eastern India. Soil and Tillage Research. 2024;236:105940. 10.1016/j.still.2023.105940.

[56] Lair GJ, Zehetner F, Khan ZH, Gerzabek MH. Phosphorus sorption–desorption in alluvial soils of a young weathering sequence at the Danube River. Geoderma. 2009;149(1):39–44. 10.1016/j.geoderma.2008.11.011.

[57] Bai J, Ye X, Jia J, Zhang G, Zhao Q, Cui B, et al. Phosphorus sorption-desorption and effects of temperature, pH and salinity on phosphorus sorption in marsh soils from coastal wetlands with different flooding conditions. Chemosphere. 2017;188:677–88. doi: 10.1016/j.chemosphere.2017.08.117 28923731

[58] Jalali M, Jalali M. Relation between various soil phosphorus extraction methods and sorption parameters in calcareous soils with different texture. Science of The Total Environment. 2016;566–567:1080–93. doi: 10.1016/j.scitotenv.2016.05.133 27297266

[59] Yang X, Wang Y, Wang X, Niu T, Abid AA, Aioub AAA, et al. Contrasting fertilization response of soil phosphorus forms and functional bacteria in two newly reclaimed vegetable soils. Science of The Total Environment. 2024;912:169479. doi: 10.1016/j.scitotenv.2023.169479 38123102

[60] Jing K, Min X, Song W, Xu D, Li X. Effect of filling materials on reconstructed soil phosphorus adsorption and desorption in mining area. Soil and Tillage Research. 2024;235:105895. 10.1016/j.still.2023.105895.

[61] Hawkins JMB, Vermeiren C, Blackwell MSA, Darch T, Granger SJ, Dunham SJ, et al. The effect of soil organic matter on long-term availability of phosphorus in soil: Evaluation in a biological P mining experiment. Geoderma. 2022;423:115965. 10.1016/j.geoderma.2022.115965.

[62] Yang J, Xin X, Zhang X, Zhong X, Yang W, Ren G, et al. Effects of soil physical and chemical properties on phosphorus adsorption-desorption in fluvo-aquic soil under conservation tillage. Soil and Tillage Research. 2023;234:105840. 10.1016/j.still.2023.105840.

[63] Daly K, Jeffrey D, Tunney H. The effect of soil type on phosphorus sorption capacity and desorption dynamics in Irish grassland soils. Soil Use and Management. 2001;17(1):12–20. 10.1111/j.1475-2743.2001.tb00003.x.

[64] Fink JR, Inda AV, Bavaresco J, Barrón V, Torrent J, Bayer C. Phosphorus adsorption and desorption in undisturbed samples from subtropical soils under conventional tillage or no-tillage. Journal of Plant Nutrition and Soil Science. 2016;179(2):198–205. 10.1002/jpln.201500017.

[65] Amadou I, Faucon M-P, Houben D. New insights into sorption and desorption of organic phosphorus on goethite, gibbsite, kaolinite and montmorillonite. Applied Geochemistry. 2022;143:105378. 10.1016/j.apgeochem.2022.105378.

[66] Fink JR, Inda AV, Bavaresco J, Barrón V, Torrent J, Bayer C. Adsorption and desorption of phosphorus in subtropical soils as affected by management system and mineralogy. Soil and Tillage Research. 2016;155:62–8. 10.1016/j.still.2015.07.017.

[67] Abboud FY, Favaretto N, Motta ACV, Barth G, Goularte GD. Phosphorus mobility and degree of saturation in oxisol under no-tillage after long-term dairy liquid manure application. Soil and Tillage Research. 2018;177:45–53. 10.1016/j.still.2017.11.014.

