# Supplementary figures and images for: Study on vertical variation characteristics of soil phosphorus adsorption and desorption in black soil region of Northeast China
Source: PLoS One. 2024 Jun 24;19(6):e0306145. doi: 10.1371/journal.pone.0306145 (PMC11195998; doi:10.1371/journal.pone.0306145)

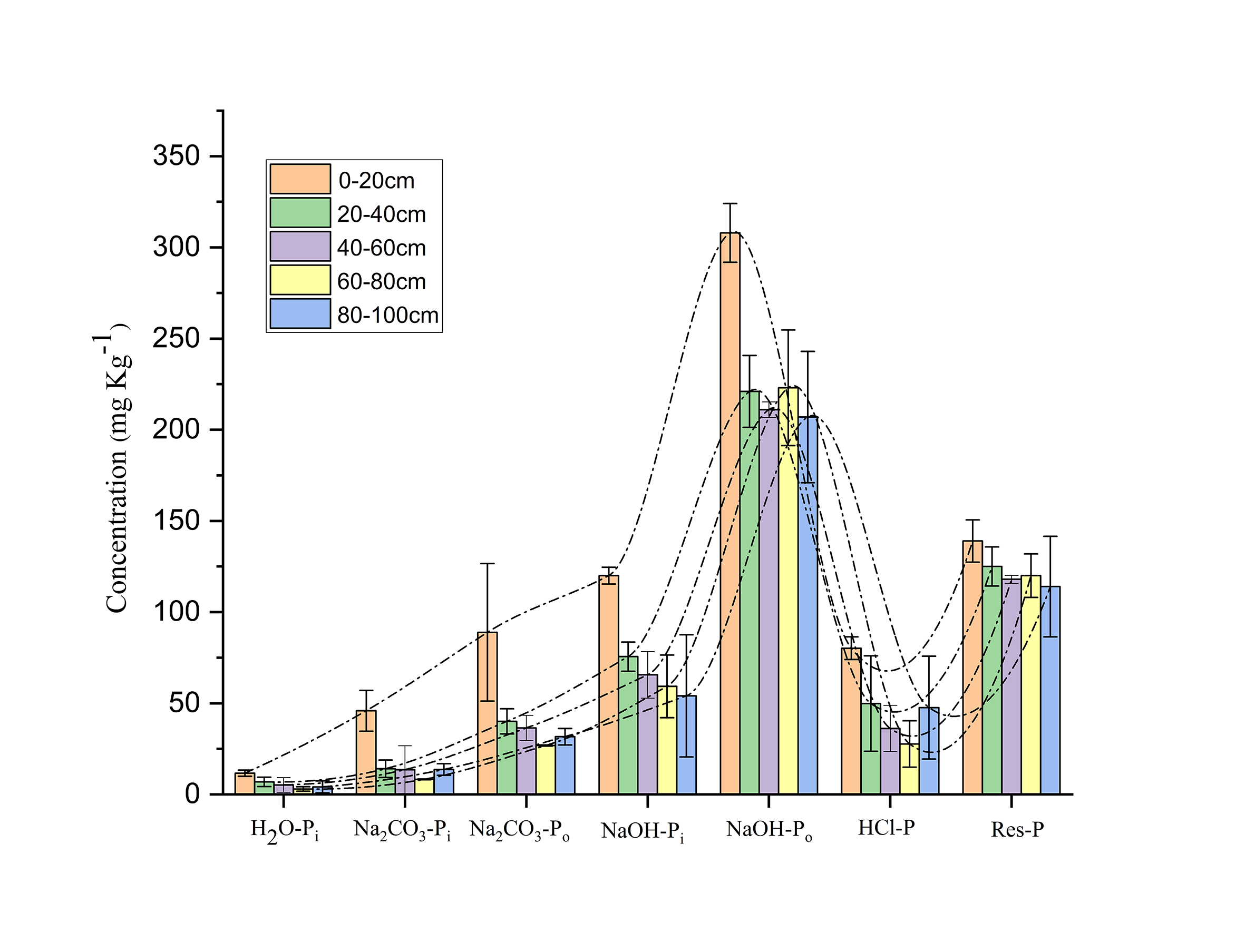

Supplement: S1 Fig — (TIF) [file pone.0306145.s001.tif]

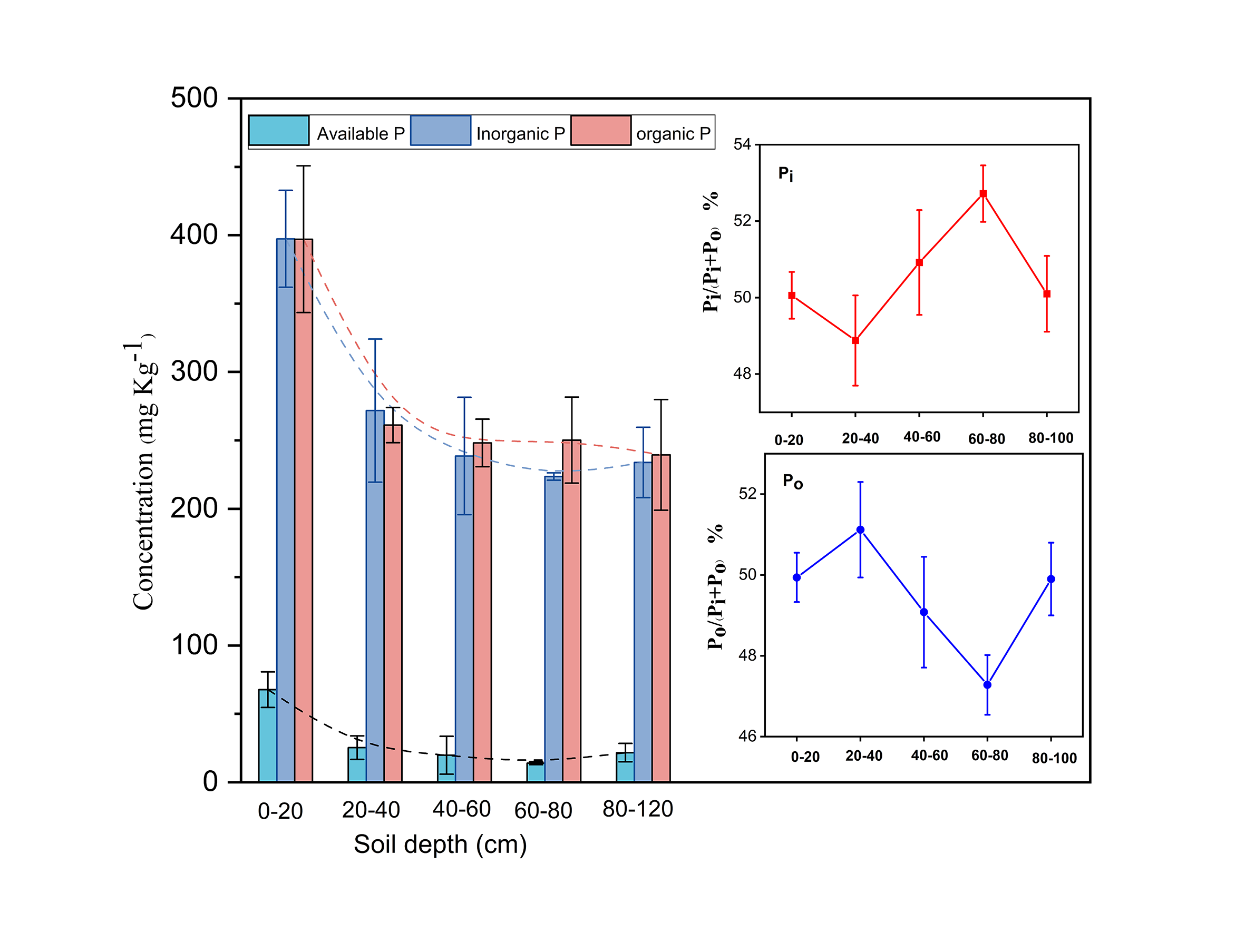

Supplement: S2 Fig — (TIF) [file pone.0306145.s002.tif]

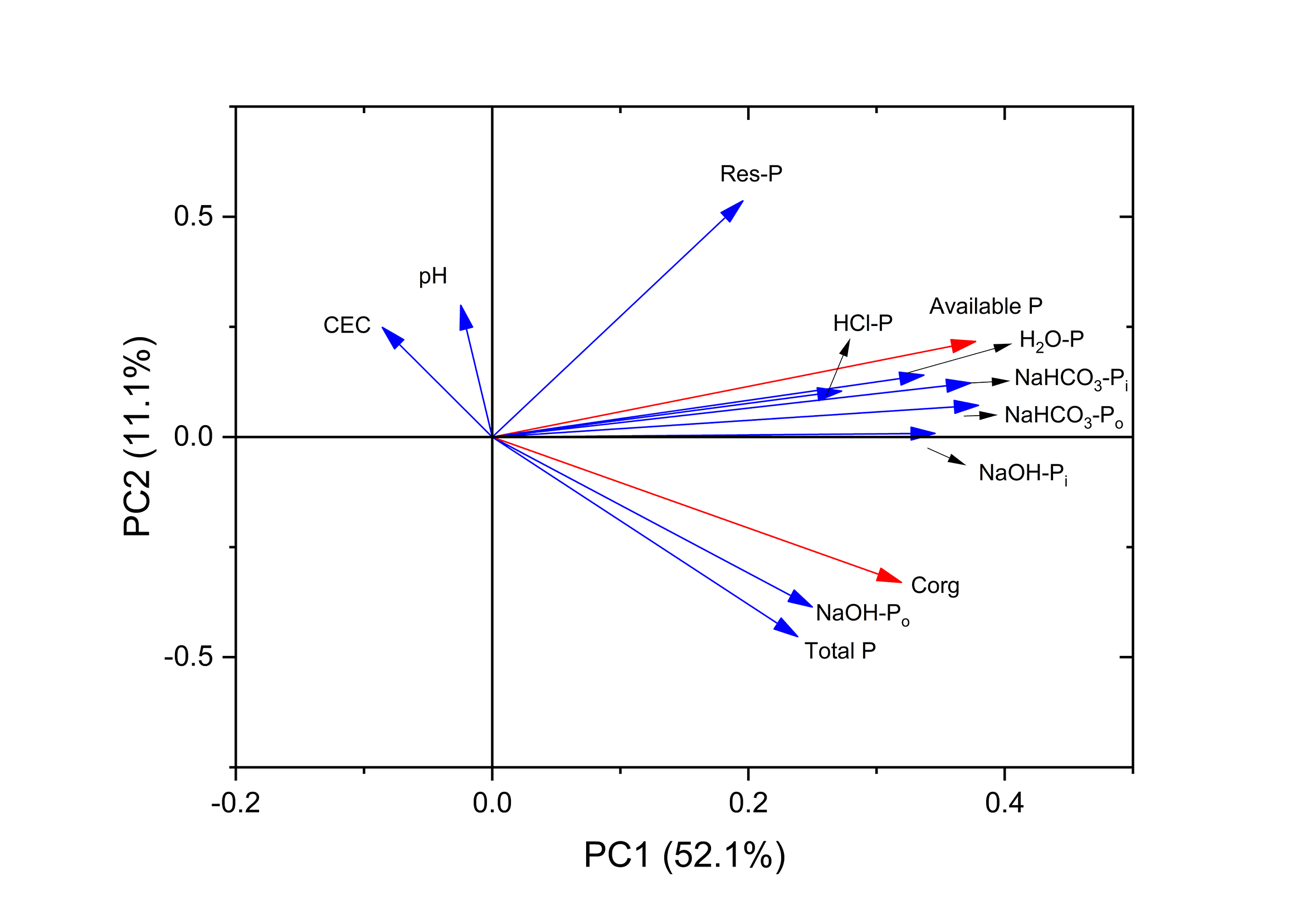

Supplement: S3 Fig — (TIF) [file pone.0306145.s003.tif]

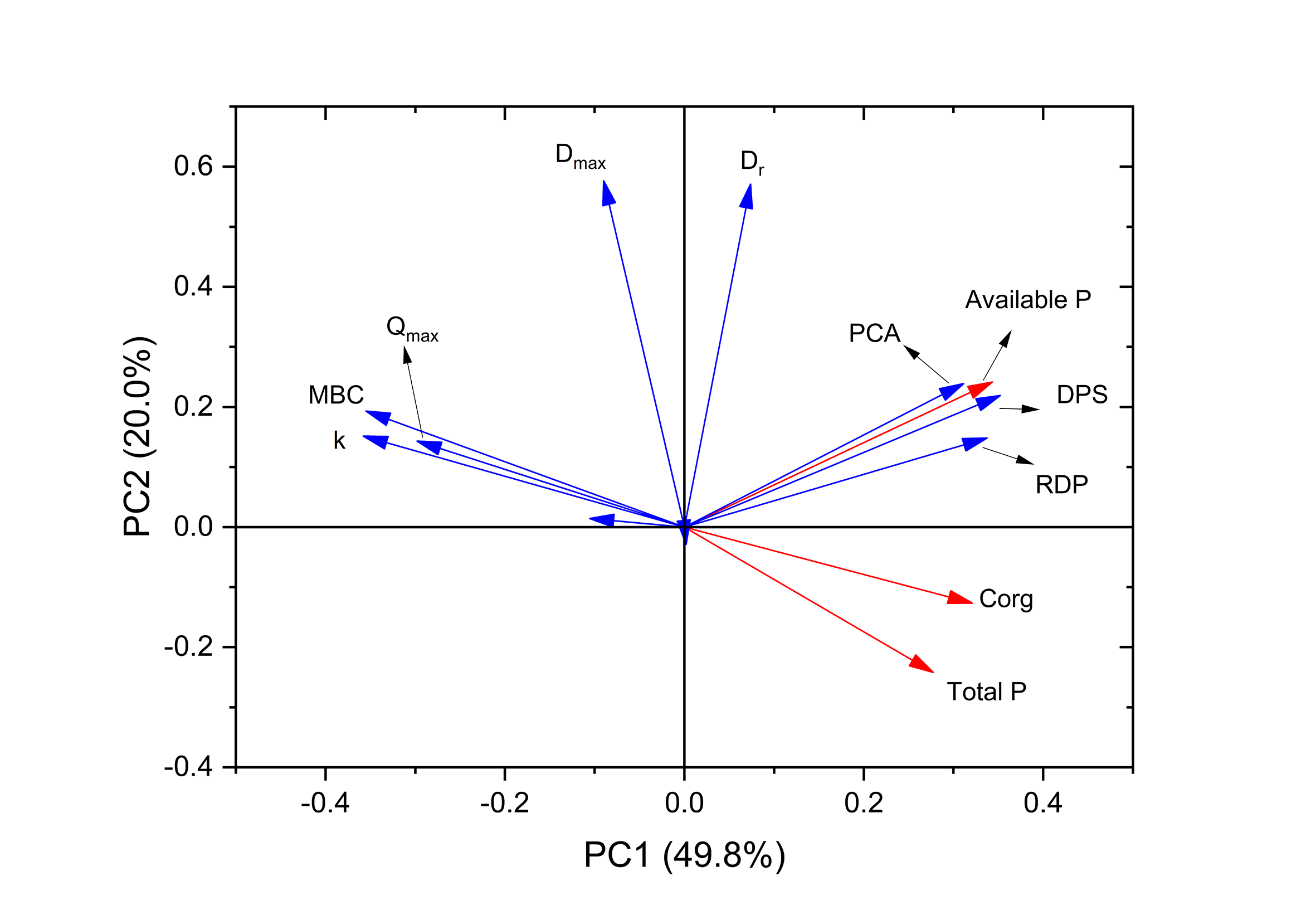

Supplement: S4 Fig — (TIF) [file pone.0306145.s004.tif]
