# Supplementary material for: Study on vertical variation characteristics of soil phosphorus adsorption and desorption in black soil region of Northeast China
Source: PLoS One. 2024 Jun 24;19(6):e0306145. doi: 10.1371/journal.pone.0306145 (PMC11195998; doi:10.1371/journal.pone.0306145)
Supplement: S1 Table — (DOC) [file pone.0306145.s005.doc]

**S1 Table** **Mechanical composition of soil in the study area (0-100 cm)**

| Soil layers  (cm) | 黏粒(%) | 粉粒(%) | 砂粒(%) | 孔隙度(%) |
| --- | --- | --- | --- | --- |
| 0-20 | 42.2±0.26  (38.5-44.1) | 43.0±0.33  (40.2-45.3) | 14.8±0.11  (13.7-15.6) | 66.2±0.38  (60.5-69.8) |
| 20-40 | 40.8±0.28  (38.1-42.7) | 31.9±0.25  (29.7-33.6) | 27.3±0.14  (26.4-28.9) | 61.5±0.34  (57.4-65.5) |
| 40-60 | 31.4±0.24  (28.5-34.2) | 29.4±0.24  (26.5-32.5) | 39.2±0.28  (37.1-42.5) | 49.7±0.34  (45.3-53.2) |
| 60-80 | 38.7±0.27  (37.2-40.3) | 32.5±0.26  (31.4-35.7) | 28.8±0.26  (27.3-30.6) | 43.5±0.29  (40.1-46.4) |
| 80-100 | 41.6±0.25  (38.5-44.2) | 37.3±0.28  (40.2-45.1) | 21.1±0.20  (20.3-23.5) | 41.8±0.31  (37.5-45.7) |

Note: The values enclosed in parentheses represent the range of concentration.
